# Volatility criteria and physicochemical properties of the promising dimethyl carbonate-gasoline blends

**DOI:** 10.1038/s41598-022-21303-3

**Published:** 2022-10-13

**Authors:** Manal Amine, Hoda A. Mohammed, Y Barakat

**Affiliations:** 1 Processes Design & Development Department, Egyptian Petroleum Research Institute, Cairo, Egypt; 2 Analysis & Evaluation Department, Egyptian Petroleum Research Institute, Cairo, Egypt

**Keywords:** Chemistry, Energy science and technology

## Abstract

Increased need for energy resources, as well as the urgent need to improve the air quality, have prompted further research to meet these challenges. Great efforts have been directed to reducing the impact of exhaust emissions. In literature, the effect of blending dimethyl carbonate (DMC) into fuel on engine performance and exhaust emissions has been investigated, and the obtained results were promising in decreasing exhaust emissions. In the present work, the effect of blending DMC into gasoline on the physicochemical properties was studied. Six fuel blends were prepared by blending base gasoline (G) with (0%, 2%, 4%, 6%, 8%, and 10%) of DMC. The volatility characteristics of the fuel blends were studied, such as the distillation curve, vapor pressure, and driveability index. The octane rating and the physicochemical properties of the fuel blends were also studied. The results of the study showed interesting findings that encourage refineries to be interested in this promising fuel additive. The results showed that the addition of DMC to gasoline has a very slight effect on the volatility of gasoline, unlike other oxygenated additives like short chain alcohols which cause a significant increase in the fuel volatility. The addition of DMC to gasoline causes an insignificant increase in the vapor pressure as the addition of 10% of DMC increases the vapor pressure by 2 kPa while it does not affect the values of T10, T50, and T90, which are the most important parameters of the distillation curve. The results also showed that its addition causes a remarkable increase in the octane rating. The RON has increased for the G-10DMC blend by about 5 points making the DMC a promising octane booster.

## Introduction

The global warming phenomenon mainly resulted from the increasing use of non-environmentally friendly sources of energy such as coal and crude oil. Petroleum derivatives (such as gasoline and diesel) used in the transportation sector represent the primary sources of increasing the greenhouse gas emissions, which greatly exacerbate the problem of global warming^1,2^. Great efforts have been made to decrease global warming. The tremendous progress that has been made in the fields of clean energy, such as wind, and solar energy, contributes greatly to reducing this phenomenon^3–5^. New sources of energy for transportation are now under investigation, such as hydrogen^6^. On the other hand, great efforts were directed to reduce exhaust emissions that result from the use of petroleum fuels, especially used for automobile engines.

Oxygenates (which are flammable organic compounds containing one or more oxygen atoms) have been used as gasoline additives mainly for two reasons, one of them is to boost octane number, and the other is to improve exhaust emissions^7,8^. Methyl tert-butyl ether (MTBE) was used as an oxygenated additive for gasoline as it has a significant impact on improving octane number and on improving air quality. However, MTBE has been banned in many countries as it has been found to contaminate groundwater. Researchers have investigated many alternatives to MTBE, such as alcohols (methanol, ethanol, butanols, etc.), ethers (ethyl tert-butyl ether, diethyl ether, etc.), and esters (methyl acetate, ethyl acetate, etc.)^9–11^.

Short chain alcohols such as methanol and ethanol are currently used in many countries as gasoline additives as they enhance the octane number and improve exhaust emissions^12–14^. Also, using such renewable oxygenates can decrease the complete dependence on petroleum oil as the main source of energy^15–18^.

Dimethyl Carbonate (DMC) has been studied as a fuel additive. DMC is considered as oxygenated compound as it contains three atoms of oxygen. It is a flammable organic solvent with the chemical formula OC(OCH_3_)_2_. It is considered as a green reagent, so it attracts more attention. It is a non-polar, non-toxic solvent with good miscibility with water and is biodegradable in the atmosphere. DMC is rated in the greenest “recommended” category according to the Solvent Selection Guide. It can be a potential replacement for many solvents, and it is usually used as a methylating agent.

DMC could be produced from the reaction of phosgene (COCl_2_) with methanol that produces methyl chloroformate, which reacts again with methanol to give DMC^19^. This method is no longer used industrially due to the high toxicity of phosgene and the corrosive HCl produced as a byproduct.

DMC can also be produced by an esterification reaction in which CO_2_ is reacted with an epoxide to yield cyclic carbonate, which is then reacted with alcohol by esterification to give DMC, but this method is not economical as it is of low yield and high cost.

DMC is primarily produced by the carbonylation processes (over 90% of global production). In this method, CO_2_ is used as a raw material to produce DMC^20,21^. DMC can be prepared easily from the direct reaction of carbon dioxide and methanol. This process is characterized by simplicity, cheapness, safety, and no intermediates production. The approach of transforming waste CO_2_ into valuable compounds is considered as a green chemical process^22^ and contributes significantly in reducing the impact of CO_2_ on climate change.

In literature, there are many works that investigated the effect of blending DMC into diesel and gasoline fuel. Most of these studies concentrated on combustion and engine performance. Zhang et al.^23^ examined the impacts of DMC on emissions and the performance of compression ignition engine. The study demonstrated that the smoke and NOx emissions were decreased. They attributed the obtained results to the high oxygen content of DMC, so its addition could enhance the oxygen content of the DMC-fuel blends, reduce the carbon-to-hydrogen ratio and reduce the aromatic fraction, which results in decreasing smoke and soot formation.

Cheung et al.^24^ investigated the impact of DMC/diesel on the emissions. The obtained data showed that the concentration of the particulate mass and the particle number of soot significantly decreased. Huang et al.^25^ investigated the impact of blending DMC with diesel on the combustion characteristics and the heat released by a direct injection diesel engine. They found that DMC leads to a slight decrease in the cetane number, the lower-heat value, and the maximum gas temperature.

Mei et al.^26^ investigated the influence of blending DMC with diesel on the combustion process and the emissions of the exhaust of a single-cylinder CI engine. They found that the density of the volumetric energy was reduced by adding DMC. They found also, that the emitted gases HC, PM, and CO reduced due to the enhancement in oxygen content in the fuel blended with DMC while NOx emissions increased. Also, the flammability of the blend was not affected by the low cetane number of DMC. Wang et al.^27^ studied the engine performance and the emissions of an engine fueled with a DMC/diesel blend. The obtained data indicated that the fuel consumption rises with the increase in the concentration of DMC in the blend, which was attributed to the low lower-heat value of DMC compared to diesel. They also found that the brake-specific fuel consumption was reduced to a slight extent. The obtained result could be caused by the enhancement in the combustion process, which was attributed to the high oxygen content of the DMC fuel blend. Also, the effective thermal efficiency improved for all blends except one containing 20% of DMC. Hence, they deduced that small concentrations of DMC can achieve better combustion while high concentrations of DMC could decrease the lower-heat value.

Gopinath et al.^28^ examined the effect of blending DMC into gasoline on the performance of an SI engine. They found that the brake thermal efficiency was improved compared to base gasoline, and there was a decrease in CO and HC emissions. They attributed the obtained results to the rise of the octane number of the blends and the optimum combustion process.

The most common drawback of using DMC is its lower heating value compared to that of hydrocarbon gasoline, and other oxygenates, which causes an increase in fuel consumption.

**Figure 1:**
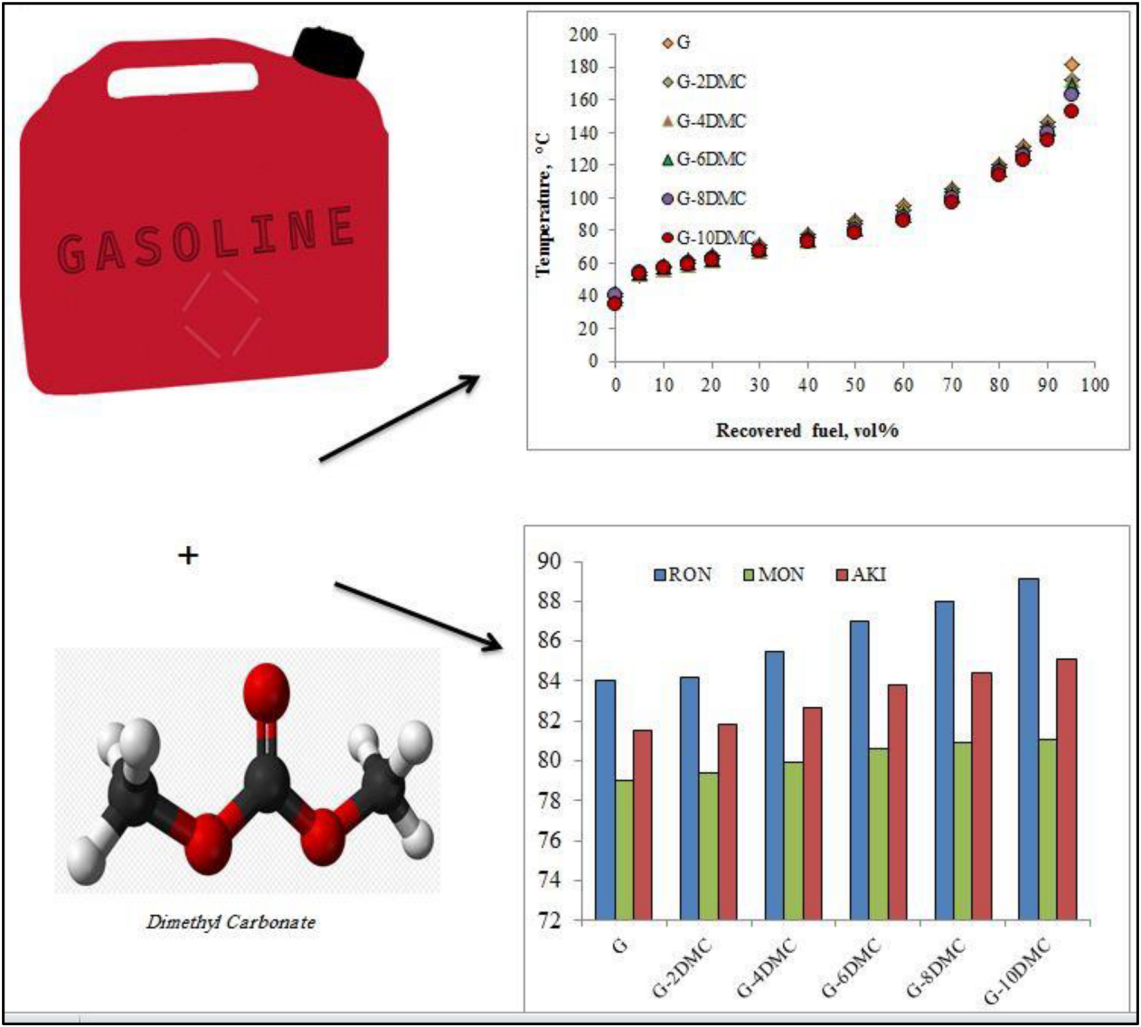
The effect of DMC on the distillation curve and the octane rating of gasoline.

Almost all the works presented in the literature were concerned with studying exhaust emissions and engine performance, and only a few were interested in the physicochemical properties of DMC/fuel blends. This work aims to examine the effect of blending DMC into gasoline fuel on the physicochemical properties, Octane rating, and the volatility criteria such as distillation curve, vapor pressure, and driveability index. Figure 1 illustrates the most important findings in the presented work.

## Materials and methods

### Fuel formulation

The hydrocarbon gasoline was brought from the Cairo Petroleum Company. The composition of the base gasoline was analyzed by gas chromatographic analysis and is given in Table 1. Dimethyl carbonate (99%) was purchased from ACROS ORGANICS Co. Absolute ethanol (99.9%) was purchased from Carlo Erpa Co. Six fuel blends (G, G-2DMC, G-4DMC, G-6DMC, G-8DMC, and G-10DMC) were formulated by mixing (0, 2, 4, 6, 8, and 10) of DMC and gasoline; respectively.

**Table 1:** Chemical composition of the base gasoline (G).

Components	wt%
Propane	0.033
i-Butane	0.422
n-Butane	2.080
i-pentane	10.504
n-Pentane	9.932
Hexanes	28.726
Benzene	2.219
Heptanes	16.560
Toluene	8.176
Octane	8.712
Ethyl-benzene	0.812
p.m-xylene	3.861
o-xylene	1.140
Nonanes	3.472
Decanes	1.867
Undecanes	0.801
Dodecanes	0.369
Tridecanes	0.143
Tetradecanes	0.077
Pentadecanes	0.035
Hexadecanes	0.021
Heptadecanes	0.016
Octadecanes	0.012
Nonadecanes	0.007
Icosanes	0.003
Total	100.000
Total aromatics	16.208
Total paraffenes	83.792

### Fuel blends characterization

The distillation profiles were given according to ASTM-D86 using STARDist Automatic Distillation unit MODEL No.913021 while the vapor pressures were measured according to ASTM-D5191 using the “Mini method”. SETAvap II, Automatic vapor pressure Tester-81000-2, was used for measuring the vapor pressure. The octane rating was measured by ZELTEX ZX-101X portable near-infrared octane –cetane analyzer. In this device, the near-infrared light that enters the sample is scattered, and part of the light beam is absorbed within the sample. The ZX-101XL measures the spectra exiting the sample. Some experiments were repeated tree times to minimize the experimental errors. Table 2 showed the accuracy of the main parameters as given by its corresponding device.

**Table 2:** Uncertainty of the parameters.

Vapor pressure	± 0.5 kPa
Distilled fuel	± 0.01 ml
RON	± 0.25
MON	± 0.12
Density	0.00005 g/cm^3^
Kinematic viscosity	± 0.35%
Sulfur content	0.002 wt%

## Results and discussion

### Volatility criteria

Volatility is one of the most important characteristics of gasoline. It is vital for performance as the fuel must vaporize first before ignition. Also, it is important to be adjusted for environmental concerns. There are many volatility criteria that differ from country to country. Gasoline fuel must meet the volatility standards approved in the country.

#### Vapor pressure

Vapor pressure is one of the most common measures of gasoline volatility. Environmental Protection Agency (EPA) regulates the gasoline vapor pressure depending on the seasons and the geographical area. The rules set by EPA aims to reduce the emitted volatile organic compounds (VOC), especially in the summertime. The vapor pressure of gasoline is very significant for the startability of the engine, so it should be formulated to be high enough in cold weather to ensure good startability and good operation. Table 3 shows that blending DMC into gasoline fuels led to an insignificant increase in the vapor pressure so there is no fear of adding DMC to gasoline as there are no problems that can occur with regard to the vapor pressure.

**Table 3:** Distillation data and volatility criteria of fuel blends.

Blend composition (ml)	G	G-2DMC	G-4DMC	G-6DMC	G-8DMC	G-10DMC
Gasoline	100	98	96	94	92	90
Dimethyl carbonate	0	2	4	6	8	10
Total	100	100	100	100	100	100
**Distillation data**						
IBP, °C	41.06	39.14	38.95	40.32	40.3	35.5
T10, °C	58.46	57.85	56.66	57.85	57.51	56.93
T20, °C	64.39	62.78	62.23	63.25	62.65	62.04
T30, °C	70.69	69.22	67.76	68.88	67.75	67.19
T40, °C	77.75	75.91	74.22	74.86	73.59	72.7
T50, °C	85.75	83.74	81.91	81.52	80.26	78.65
T60, °C	95.02	92.53	91.26	90.06	88.22	85.89
T70, °C	105.87	103.62	102.88	101.8	100.25	97.12
T80, °C	120.63	118.5	117.96	117.49	115.77	113.35
T90, °C	146.46	143.56	143.14	142.57	139.74	135.18
T95, °C	181.45	172.07	171.8	168.77	162.53	153.23
FBP, °C	202.25	197.82	197.74	194.59	194.2	187.91
Vapor pressure (kPa)	58.1	59.3	59.3	59.5	59.5	59.9
E70, vol%	31	33	33	33	33	34
E100, vol%	68	67	68	68	70	72
E150, vol%	93	92	92	92	92	94
DI	491.4	479.87	473.86	473.8	466.7	456.5
T_(v/l=20)_, °C	59.56	58.53	58.157	58.2	57.98	57.45

#### Distillation curve

One of the most important volatility criteria of gasoline is the distillation curve, which is drawn in this work according to ASTM-D86. It is a relation between the recovered volume of the fuel distilled and the distillation temperature. Some important characteristics are derived from the data obtained from this curve, such as driveability index and Temperature for Vapor–Liquid Ratio of 20 (T_V/L=20_). The strong impact of the distillation curve on the engine performance has led to setting regulations to control this curve. T10, T50, and T90 are the most important temperature degrees recorded from the distillation curve, and they have restricted limits according to ASTM-D4814. They represent the temperature degrees at which 10, 50, and 90% of the fuel is distilled; respectively. T10 is important to ensure good startability, especially in cold weather. It also must be adjusted to control the evaporative emissions and vapor lock, especially in hot weather. T50 represents the Midrange volatility that must be controlled to ensure a balance between low and high boiling gasoline constituents. Controlling T50 ensures engine warming-up, good acceleration and avoiding ice formation. T90 is important for fuel economy^29^.

**Figure 2:**
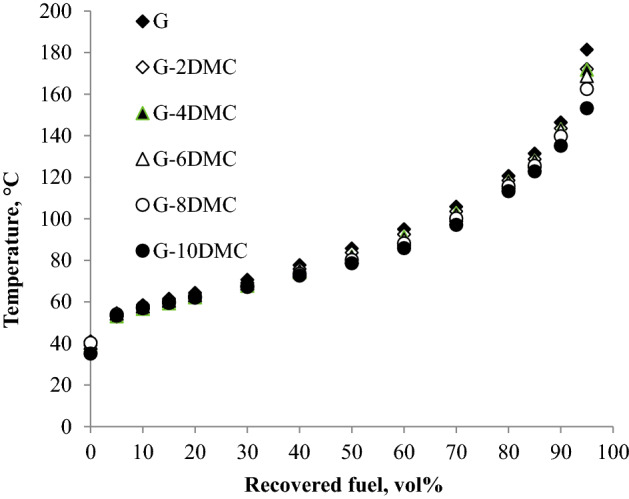
The impact of blending DMC into gasoline on the distillation curve.

**Figure 3:**
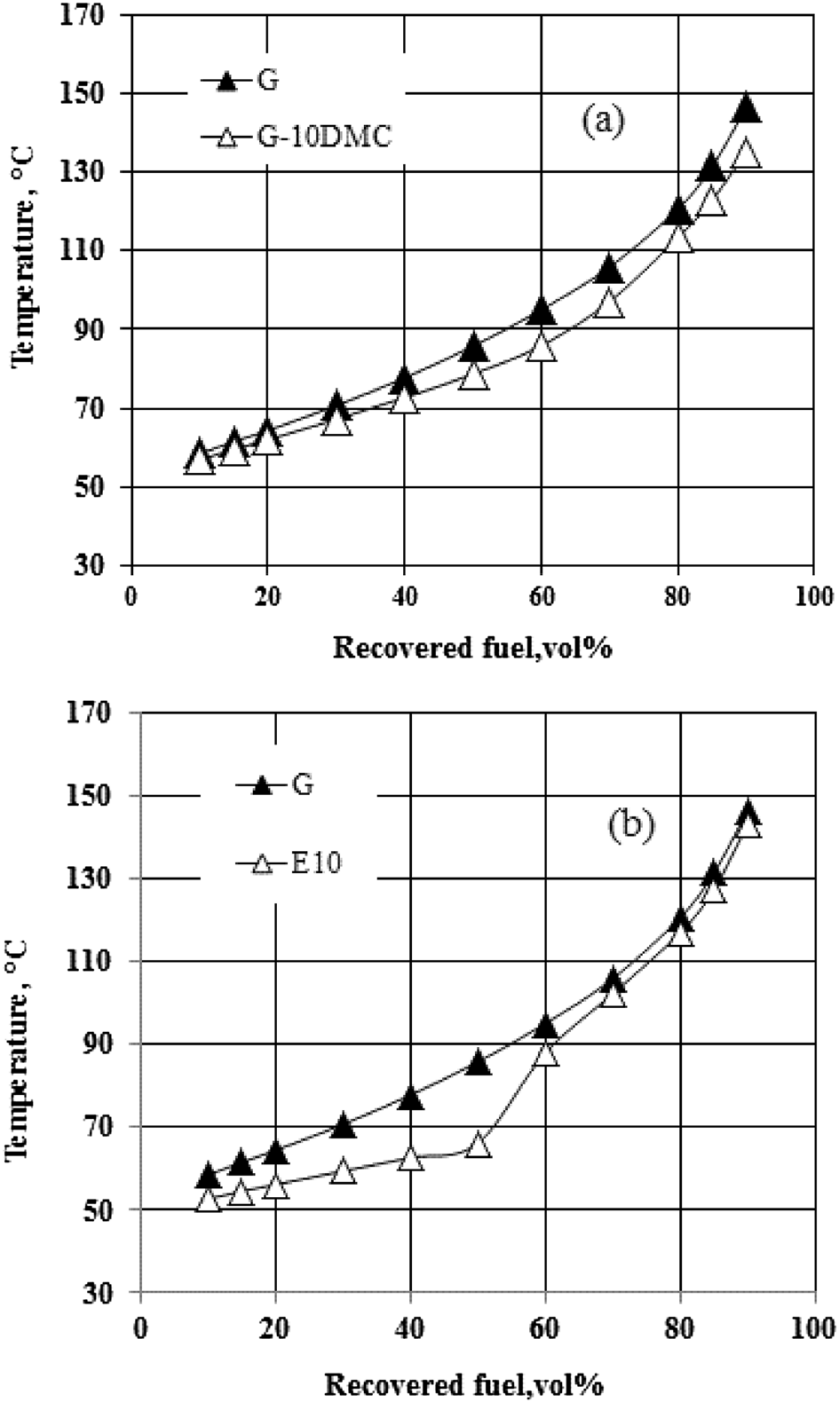
Illustrates the difference between the impact of blending 10% of DMC (**a**) and the impact of blending 10% of ethanol (**b**) on the distillation curve of gasoline.

Figure 2 clearly shows that DMC does not significantly affect the distillation curve. Looking at the front end of the curves (up to 40% of fuel distilled) in Fig. 2, the curves are almost stacked on each other, which mean that the addition of DMC does not affect the lighter fraction of gasoline. Based on the obtained results we can assume that the addition of DMC does not affect the startability of the engine and does not increase the evaporative emissions as ethanol does. Only about 5 vol% increase in the fuel distilled at T50 for blend containing 10% of DMC, and all the samples meet the requirements of the gasoline volatility approved by ASTM-D4814 as presented in Table 4. En 228 set other parameters that control the distillation curve, such as E70, E100, and E150 which represent the volume percent of the fuel distilled at temperature degrees 70, 100, and 150 °C, respectively. E70, E100, and E150 of the fuel blends are shown in Table 3. For more clarification, an ethanol-gasoline blend containing 10% of absolute ethanol was distilled for comparison. Figure 3 shows that the increase in the fuel distilled does not exceed 6% (v/v) for the 10% (v/v) DMC-gasoline blend at 70 °C. By comparing the result of the gasoline blend containing 10% of DMC with that of the ethanol-gasoline blend; we found that E70 was increased by about 20% (v/v) for the 10% ethanol addition, as shown in Fig. 3. This result indicates that DMC does not cause any problems in the volatility of gasoline as ethanol and other oxygenated additives as shown in Table 6.

**Table 4:** Standards of the volatility parameters.

Parameter (units)	Standard	Minimum	Maximum
RVP (kPa)	ASTM 4814	–	79
T10, °C	ASTM 4814	–	60
T50, °C	ASTM 4814	77	116
T90, °C	ASTM 4814	–	185
RVP(kPa)	EN228	50	80
E70% (v/v)	EN228	22	50
E100% (v/v)	EN228	46	71
E150% (v/v)	EN228	75	–
DI	ASTM 4814	375	610
T_(V/L=20)_, °C	ASTM 4814	35	55

#### Driveability index (DI)

Driveability index is a term expressing one of the volatility parameters of gasoline. It is a relationship developed by a combination of various points of the distillation curve to describe the engine driveability. The driveability index can be calculated from the following correlation:

$${\text{DI}} = {1}.{5} \times {\text{T1}}0 + {3}.0 \times {\text{T5}}0 + {1}.0 \times {\text{T9}}0.$$

**Figure 4:**
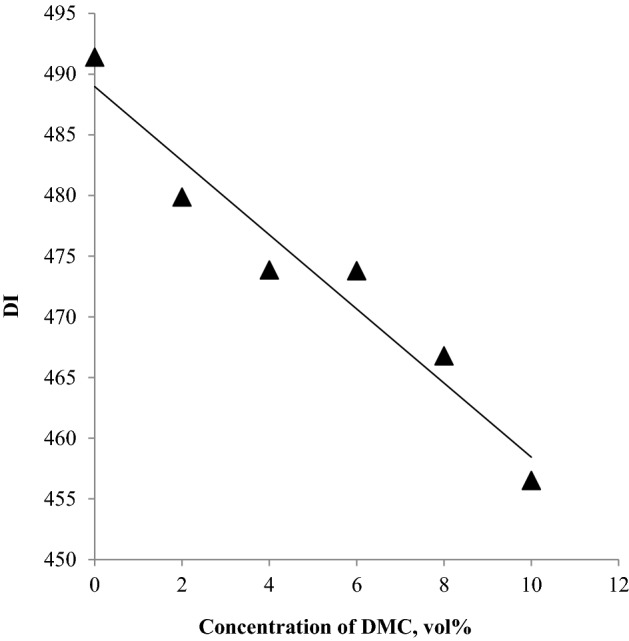
The impact of blending DMC into gasoline on the DI.

ASTM-D4814 set limits to drivability index specified between 375 and 610 °C. As shown in Fig. 4, DMC addition decreases the DI, but all the results are within the normal range defined by ASTM-D4814.

#### Temperature of the vapor liquid ratio of 20

Vapor pressure alone is not enough to control the vaporization of gasoline. Another parameter expressing the gasoline vaporization is the temperature at which the vapor-to-liquid ratio equals 20. This temperature is important to be adjusted to avoid the tendency of the fuel to form a vapor lock in the fuel path inside the engine. The lower the temperature at which the vapor to liquid ratio equals 20, the higher the fuel tends to form a vapor lock. This temperature could be estimated according to the following equation^30^:

$${\text{T}}_{{{\text{V}}/{\text{L}} = {2}0}} = \, \left[ {{52}.{47 }{-} \, 0.{33}\left( {{\text{VP}}} \right)} \right] \, + \, 0.{2}0{\text{ T}}_{{{1}0}} + \, 0.{\text{17 T}}_{{{5}0}} .$$

This equation is approved by ASTM-D4814 where VP in kPa, T10, and T50 in °C. There are six classes for vapor lock protection, and the normal minimum temperature specified for V/L = 20 is 35–54 °C (95–129 °F).

**Figure 5:**
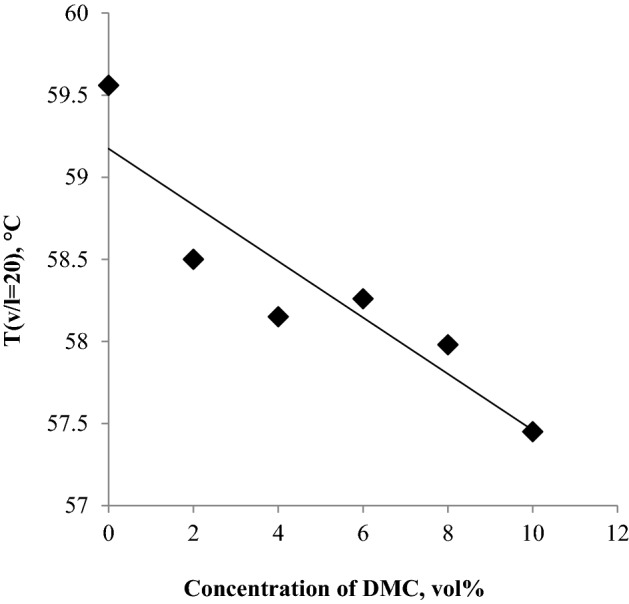
The impact of blending DMC into gasoline on the T(v/l = 20).

For the blends under investigation, as shown in Fig. 5 the addition of DMC slightly decreases the temperatures of the vapor liquid ratio of 20, and all the obtained results exceed the minimum temperature specified by ASTM-D4814, so there is no fear of vapor lock formation in the fuel system specially when using a DMC-gasoline blend in hot weather.

### Density

**Figure 6:**
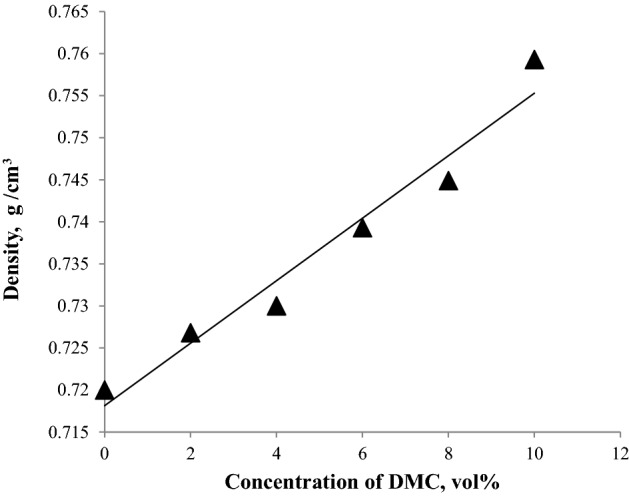
The impact of blending DMC into gasoline on the density.

**Figure 7:**
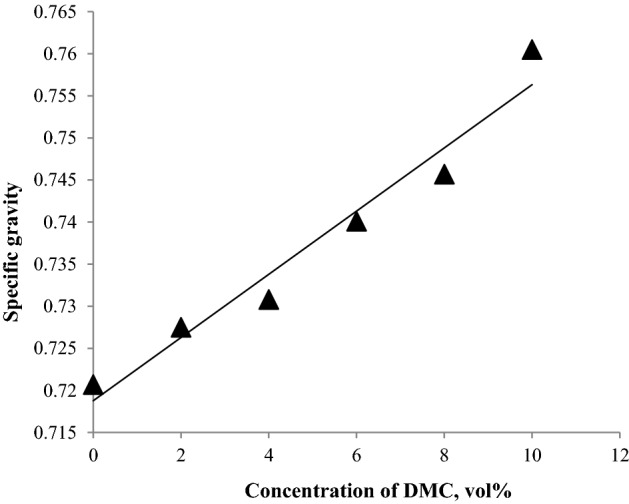
The impact of blending DMC into gasoline on the specific gravity.

The density of the fuel is important as it correlates to the volumetric energy content of the fuel. As the density of the fuel increases, the volumetric energy content increases. Changes in the density affect the quantity of fuel that is injected into the combustion chamber during each cycle, altering the ideal air–fuel burning ratio. In the case of gasoline adulterated with a more dense additive, the burning will be incomplete, resulting in the emission of pollutants into the atmosphere^31^. ASTM-D4814 does not put limits on the fuel density as its measurement depends on other physical and chemical parameters. Density is measured at a specific temperature (15.56 °C), and the fuel is not usually sold at the specified temperature; Fig. 6 shows that DMC causes an increase in the density of the fuel blends. Specific gravity or relative density is usually used rather than absolute density. It is defined as the ratio of the mass of a certain volume of fuel to the mass of the same volume of water at the same temperature. Most gasoline formulations usually gave specific gravities between 0.70 and 0.78 at 15.6 °C. Figure 7 shows that DMC causes an increase in the specific gravities, but all the fuel blends gave specific gravities within the normal range^32^.

### Calorific value

**Figure 8:**
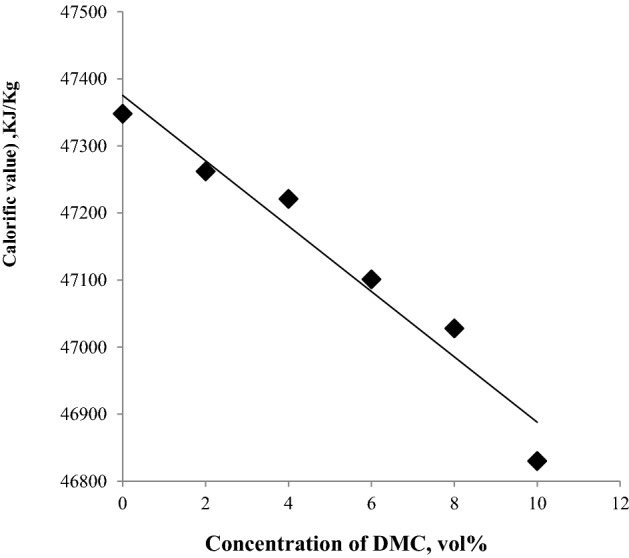
The impact of blending DMC into gasoline on the calorific value**.**

Calorific value or heating value measures the energy content that is released in the form of heat when a substance is oxidized in oxygen. Table 5 comprises the gross and net calorific values of the tested blends. Compounds with the highest hydrogen carbon ratio give the highest calorific value, so for compounds containing the same carbon numbers, paraffins are higher in heating value, followed by naphthenes, then aromatics^33^. For oxygenated compounds, the hydrogen carbon ratio is lower than that of the compounds containing the same number of carbon atoms, so the calorific values of these compounds are expected to be lower. When oxygenated compounds are mixed with hydrocarbon fuel, the calorific value of the oxygenated hydrocarbon blend is expected to be lower than the net hydrocarbon fuel. DMC was found to decrease the calorific value, as indicated in Fig. 8. In comparing the calorific values of gasoline fuel containing 10% of ethanol from literature and that of gasoline fuel containing 10% DMC from the present work, we found that The calorific value of the fuel containing ethanol decreased from 44.40 to 44.22 MJ/kg^34^ while the calorific value of gasoline containing DMC decreased from 44.148 to 43.761 MJ/kg. The decrease in the calorific value in the case of DMC can be attributed to the fact that DMC has more oxygen atoms and a lower hydrogen-to-carbon ratio than ethanol. From these results we can assume that the fuel consumption will increase in the case of using a DMC-gasoline blend.

**Table 5:** Physicochemical properties of the fuel blends.

Test		Standard test method	G	G-2DMC	G-4DMC	G-6DMC	G-8DMC	G-10DMC
Density at 15.56 °C, g/cm^3^		ASTM D-4052	0.7200	0.7268	0.7300	0.7393	0.7449	0.7593
Specific gravity			0.7207	0.7275	0.7308	0.7401	0.7457	0.7605
API gravity			64.83	63.01	62.13	59.70	58.26	54.68
Kinematic viscosity at 30 °C, cSt		ASTM D-445	0.3913	0.3879	0.3790	0.3954	0.3849	0.3683
Flash point, °C		ASTM D-56	< − 27	< − 27	< − 27	< − 27	< − 27	< − 27
Fire point, °C			< − 27	< − 27	< − 27	< − 27	< − 27	< − 27
Heating value (calorific value), kJ/kg	Gross	ASTM D-240	47,348	47,262	47,221	47,101	47,028	46,830
	Net		44,148	44,083	44,052	43,962	43,906	43,761
Water content, mg/kg		ASTM D-6304	62	73	98	110	120	220
Sulfur content, mass%		ASTM D-4294	0.0156	0.0145	0.0139	0.0140	0.0137	0.0177
Ash content, wt %		ASTM D-482	Nil	Nil	Nil	Nil	Nil	Nil

### Octane rating and knock resistance

The resistance of the fuel for knocking is the ability of the fuel not to spontaneously ignite under the influence of high compression. Octane rating is critical for the efficiency and performance of the engine. Research octane number (RON) and Motor octane number (MON) are two different testing methods for measuring octane rating. RON is a measure of the fuel resistance for knocking at mild conditions (low temperature and speed) while MON is a measure of the fuel resistance under severe conditions (high temperature and speed). The average of RON and MON represents the antiknock index (AKI). The gasoline composition controls the octane rating^8,35^. Straight-run gasoline usually has an octane number of about 70. Aromatics and highly branched compounds boost the octane number^36,37^. The olefin content in gasoline affects the drivability as the difference between RON and MON is greater than that of paraffins and aromatics, so the gasoline is formulated in such a way as to ensure good drivability by setting limits to the olefin content in gasoline. Also, the environmental concerns are important reasons to set limits for olefin content^38,39^.

**Figure 9:**
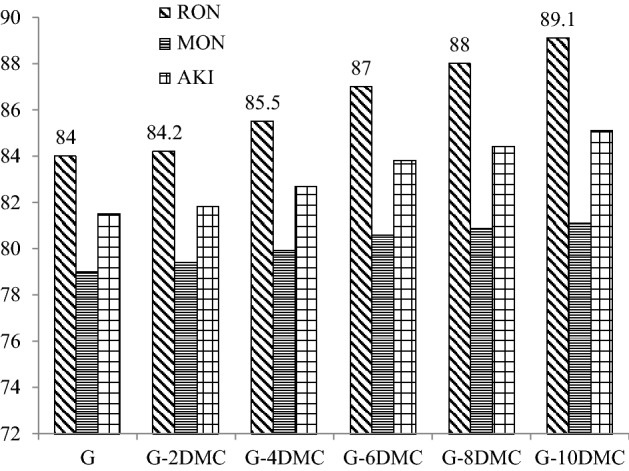
The impact of blending DMC into gasoline on the octane rating.

Figure 9 shows how the addition of DMC participates in increasing the octane rating of gasoline-DMC blends. The RON was increased for the G-10DMC blend by about 5 points which is higher than the effect of the addition of 10% of ethyl-tertbutyl ether (ETBE) and methyl tert-butyl ether (MTBE) to gasoline^40^, while its effect on RON is to some extent similar to that of ethanol^41^. The improvement of octane rating leads to enhancing engine efficiency^42^. The Figure also shows that DMC increases the MON but less than the increase in RON. AKI was found to be considerably increased, which could improve engine efficiency. Table 6 showed a comparison between some common oxygenates and DMC. The results of the comparison make DMC a promising gasoline additive for which refinery companies should consider this compound as an octane booster and prioritize it for further investigations.

**Table 6:** Effect of DMC on the change in some parameters of gasoline in comparison with other oxygenates.

Oxygenates (vol%)	Characteristics														
	VP, kPa		Ref.	T10, ºC		Ref.	T50, ºC		Ref.	RON		Ref.	Density, g/cm^3^		Ref.
	(B)	(A)		(B)	(A)		(B)	(A)		(B)	(A)		(B)	(A)	
Methanol (10%)	59.2	83	^31^	68.4	52	^31^	124.6	112	^31^	85.3	88	^31^	0.7682	0.774	^31^
Ethanol(10%)	59.6	63.85	^14^	58.46	52.65	This work	85.75	66.06	This work	93.2	97.1	^13^	0.74	0.7396	^13^
MTBE(10%)	59.2	64	^31^	68.4	62	^31^	124.6	118	^31^	85.3	87.3	^31^	0.7682	0.764	^31^
TAA (10%)	59.2	65	^31^	68.4	72	^31^	124.6	125	^31^	85.3	90.2	^31^	0.7682	0.7742	^31^
TBA (10%)	59.2	62	^31^	68.4	60	^31^	124.6	112	^31^	85.3	89.9	^31^	0.7682	0.7722	^31^
DMC (10%)	58.1	59.9	This work	58.75	57.85	This work	85.75	83.74	This work	84	89.1	This work	0.7200	0.7593	This work

*(A)* after addition, *(B)* before addition, *Ref.* reference.

## Conclusion

Discussion of the results of the experiments allows us to deduce the following conclusions. The values of the volatility criteria of dimethyl carbonate-gasoline blends that comprise vapor pressure, T10, T50, T90, E70, E100, and E150 are almost unchanged from those of base gasoline. Also, it was found that blending DMC into gasoline significantly improves the octane rating of the fuel blends as the addition of 10 vol% of DMC increases the RON by about 5 points. DMC could be a promising environmentally friendly oxygenated octane booster additive for gasoline fuel without worrying about changing the volatility characteristics.

## Data Availability

All data generated or analyzed during this study are included in this published article.

## Abbreviations

AKI: Antiknock index
API: American Petroleum Institute
ASTM: American standard for testing and materials
CO: Carbon monoxide
CO2: Carbon dioxide
DI: Driveability index
DMC: Dimethyl carbonate
E10: Gasoline–ethanol blend containing 10% ethanol
E100: Volumetric fraction of evaporated gasoline at 100 °C
E150: Volumetric fraction of evaporated gasoline at 150 °C
E70: Volumetric fraction of evaporated gasoline at 70 °C
En: European standard
EPA: European protection agency
ETBE: Ethyl tert-butyl ether
FBP: Final boiling point
G: Base gasoline
HC: Hydrocarbon
HCl: Hydrochloric acid
IBP: Initial boiling point
MON: Motor octane number
MTBE: Methyl-tert-butyl ether
RON: Research octane number
RVP: Reid vapor pressure
T(V/L = 20): Temperature at which ratio of vapor to liquid equal 20
T10: Temperature at 10% of the fuel is distilled
T50: Temperature at 50% of the fuel is distilled
T90: Temperature at 90% of the fuel is distilled
TAA: Tert-amyl alcohol
TBA: Tert-butyl alcohol
VOC: Volatile organic compounds
VP: Vapor pressure
